# Giant Congenital Melanocytic Nevi and Neurocutaneous Melanosis

**DOI:** 10.1155/2015/545603

**Published:** 2015-02-04

**Authors:** Catarina Araújo, Cristina Resende, Francisco Pardal, Celeste Brito

**Affiliations:** ^1^Dermatology Department, Hospital de Braga, Sete Fontes, São Victor, 4710-243 Braga, Portugal; ^2^Pathological Anatomy Department, Hospital de Braga, Sete Fontes, São Victor, 4710-243 Braga, Portugal

## Abstract

*Introduction*. The major medical concern with giant congenital melanocytic nevi CMN is high risk of developing cutaneous melanoma, leptomeningeal melanoma, and neurocutaneous melanocytosis. *Case Report*. A 30-year-old woman with a giant congenital melanocytic nevus covering nearly the entire right thoracodorsal region and multiple disseminated melanocytic nevi presented with neurological symptoms. Cerebral magnetic resonance imaging revealed a large expansive lesion in the left frontal region. Postsurgically pathological diagnosis revealed characteristics of melanoma. Immunohistochemical examination showed S100(+), HMB45(+), MelanA(+), and MiTF(+). She received radiotherapy with temozolomide followed by two more chemotherapy cycles with temozolomide. She followed a rapidly progressive course, reflecting widespread leptomeningeal infiltration, and she died of multiorgan failure seven months after diagnosis of cerebral melanoma. *Discussion*. This patient was diagnosed as having a neurocutaneous melanosis with malignant widespread leptomeningeal infiltration. Diffuse spinal involvement is unusual and is described in only another patient.

## 1. Introduction

Congenital melanocytic nevi (CMN) are nevi that are present at birth or arise within the first few weeks of life [1]. CMN vary in size from small to very large or giant (GCMN). These GCMN are commonly associated with benign melanocytic growths within the substance of the lesion, termed “proliferative nodules.” In addition to proliferative nodules, GCMN are often associated with “satellite nevi” [2]. These are smaller CMN that are present at birth or arise months to years later [3]. The major medical concern with giant CMN is high risk of developing cutaneous melanoma, leptomeningeal melanoma, and neurocutaneous melanocytosis.

## 2. Case Presentation

**Figure 1:**
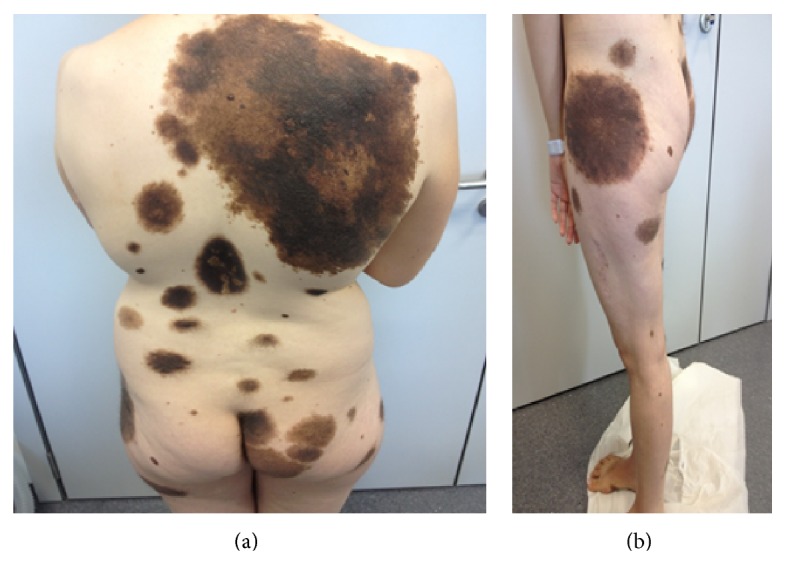
Giant black-brown pigmented nevus, one covering nearly the entire right thoracodorsal region and the other on the left thigh. Note also satellite nevi on the trunk and extremities.

A 30-year-old woman presented with impaired vision, dizziness, and headache for one week prior to admission. Physical examination revealed many brownish-black nevi on the trunk, including a giant one covering nearly the entire right thoracodorsal region (32 cm × 20 cm) and another on the left thigh (25 × 18 cm) (Figure 1). Another cephalic patch (5 cm × 4 cm) was situated on the frontal region and many brownish-black nevi on the lower limb some with abundant hair that appeared during the first years of life. Most nevi were irregular but showed no clinical signs of malignancy.

The family history was negative for congenital nevi or melanoma. She was irregularly examined by pediatric. Her early neurodevelopment had been normal although she had some learning disabilities.

**Figure 2:**
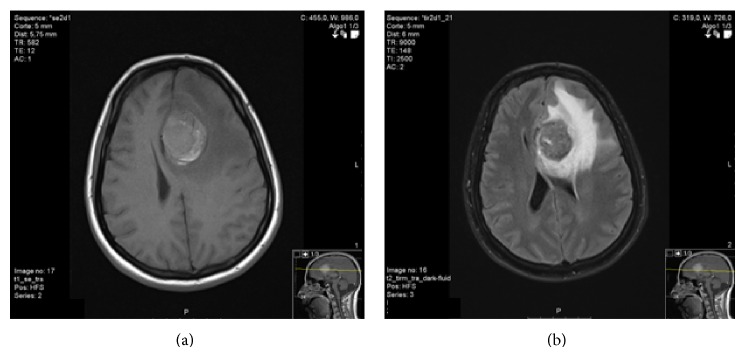
Cerebral magnetic resonance imaging revealing a large expansive lesion in the left frontal region. The lesion is heterogeneous on T1 with iso- and hyperintense areas, hypointense on FLAIR, and heterogeneous and markedly hypointense on T2.

A computed tomography (CT) scan of the head showed a hemorrhagic lesion on the medial aspect of the left frontal lobe, about 4.7 cm in maximum diameter. That was associated with parenchymal edema causing effacement of cortical sulci and anterior horns of the lateral ventricles. Cerebral magnetic resonance imaging (MRI) revealed a large expansive lesion in the left frontal region associated with hemorrhagic component showing rounded morphology and relatively well-defined contours, but with some papillomatous projections. This lesion was heterogeneous on T1 with iso- and hyperintense areas, hypointense on FLAIR, and heterogeneous and markedly hypointense on T2 (Figure 2). After contrast there was an intense uptake, inducing important lesion vascularity.

She underwent surgery by bicoronal frontal craniotomy and despite identification of the lesion (dark, very haemorrhagic) the excision was incomplete.

**Figure 3:**
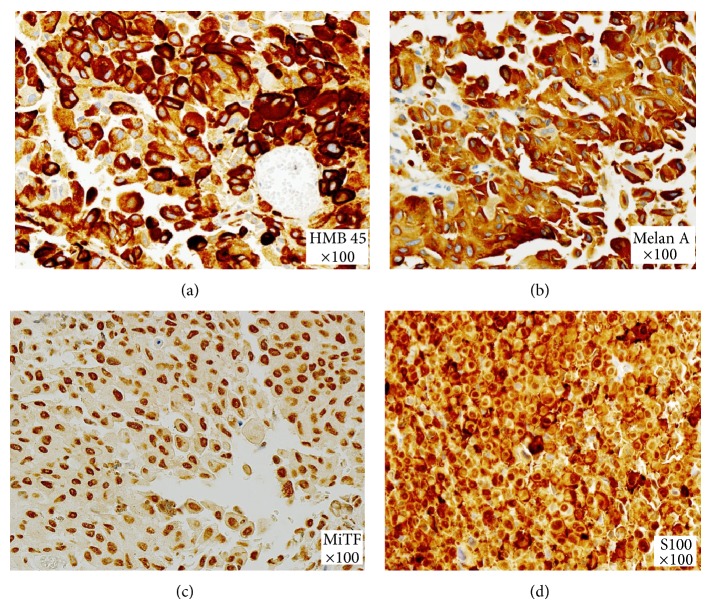
Marked cellular pleomorphism and numerous mitotic figures. Immunohistochemical examination showed S100(+), HMB45(+), MelanA(+), and MiTF(+).

Postsurgically pathological diagnosis showed brain involvement of neoplastic cells with characteristics of melanoma (Figure 3). Histologic examination revealed brain tissue widely surrounded by thick cell neoplasia, comprising nests of cells large and bulky presenting irregular nuclei of coarse chromatin and prominent and eosinophilic nucleoli. The cytoplasm presented large, dense, and slightly eosinophilic, occasionally containing melanin pigment. The neoplastic cells showed marked cellular pleomorphism. It was identified numerous mitotic figures. Immunohistochemical examination exhibited S100(+), HMB45(+), MelanA(+), and MiTF(+).

In order to find possible origin of the lesion at other sites, thoracoabdominal-pelvic computed tomography (CT) scan was done, but no lesions were detected.

She received radiotherapy (the radiation dose was 40 Gy given in 16 fractions of 2.5 Gy each) with temozolomide followed by more two chemotherapy cycles with temozolomide 200 mg/m^2^/day (for 5 days).

Six months later she was hospitalized for urinary retention. Another thoracoabdominal-pelvic computed tomography (CT) scan was done, but no lesions could be detected. Cerebral magnetic resonance imaging (MRI) revealed presence of uptake in frontal sulci corresponding to infiltration leptomeningeal tumor.

**Figure 4:**
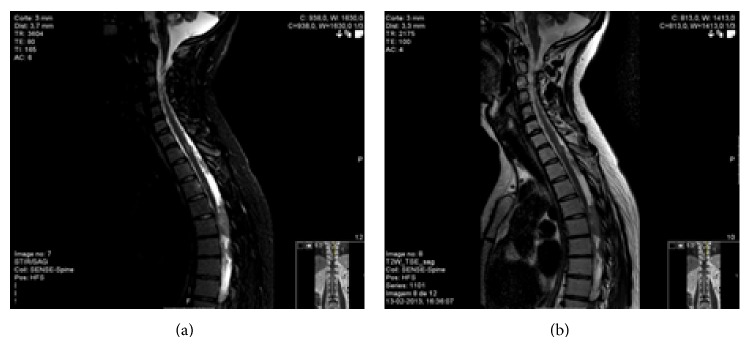
Reflecting widespread leptomeningeal infiltration, contrast capture was documented in the subarachnoid space from the level of C2-C3.

Spinal cord magnetic resonance imaging (Figure 4) exposed an extensive metastasis throughout the subarachnoid space, which extended from D4 with metastatic clusters hyperintense on T1, hyperintense on STIR, and iso/hyperintense on T2, capturing intense and homogeneous contrast. These injuries involved the conus medullaris and extended along the roots of the cauda equina depositing itself as nodule formation and metastases were observed at the inferior end of the dural sac. Reflecting widespread leptomeningeal infiltration, contrast capture was documented in the subarachnoid space from the level of C2-C3.

The patient was treated with three-dimensional computerized planning dosimetry at a dose of 30 Gy in 15 fractions to the neuraxis. Gradually she had worsening of clinical symptoms with flaccid paraplegia and sphincter injury with neurogenic bladder and constipation and she died of multiorgan failure seven months after diagnosis of melanoma.

## 3. Discussion

The existing data regarding the risk of neuromelanosis associated with CMN are imperfect but suggest a convincing link (range from 2.5% to 45%). Neurocutaneous melanosis (NCM) is neuromelanosis associated with CMN [4–9].

Neuromelanosis which relates to a congenital error in the morphogenesis of the embryonic ectoderm describes melanocytic proliferation (benign or malignant and nodular or diffuse) within the leptomeninges and brain parenchyma [4, 5, 10]. NCM can affect the amygdala, cerebrum, cerebellum, pons, medulla, and spinal cord (20%) [9, 11, 12].

In 1991, Kadonaga and Frieden [13] reviewed the literature on neurocutaneous melanosis and redefined it as follows: (1) the presence of large (more than 20 cm) and/or multiple (more than three) congenital melanocytic nevi (CMN) in association with meningeal melanosis or melanoma; (2) no evidence of cutaneous melanoma, except in patients in whom the examined areas of the meningeal lesions are histologically benign; and (3) no evidence of meningeal melanoma, except in patients in whom the examined areas of the cutaneous lesions are histologically benign.

Patients with neuromelanosis may be symptomatic or asymptomatic. There seem to be two peak ages for presentation of complications from NCM. The first peak, which represents the majority of patients, occurs before 3 years of age and those who become symptomatic are associated with increased intracranial pressure and include seizures, hydrocephalus, cranial nerve palsy, hemiparesis, and developmental delays. The other peak occurs during the second to third decades of life [6, 13, 14]. Delayed presentation in older children, adolescents, and adults has also been reported, usually with symptoms such as headaches or neuropsychiatric manifestations [15].

Suggested risk factors for NCM have been reported to be the presence of a GCMN, male sex, satellite nevi or multiple CMN, and head, neck, or posterior midline location [14–18].

Serial MRI scans may be helpful in monitoring asymptomatic patients who have a positive baseline MRI with or without signs and symptoms of CNS involvement [19]. The most common finding in this group was the T1-signal shortening compatible with melanin deposits in the infratentorial structures [20]. The FLAIR findings: leptomeningeal hyperintensity has been also described [21]. However, the routine MRI examination in these patients is debated as the sensitivity of MR imaging has permitted detection of melanosis in about 25% asymptomatic infants [19]. Other reports of metastatic melanoma also fail to demonstrate the characteristic signal intensity on MR images [22].

Neurological symptoms were a sign of poor prognosis and, in this case, the first sign of disease progression. Our patient developed a very rapid progression of the disease, with evidence of increasing extent of leptomeningeal enhancement in the brain and cord on the second image. The first appearance of these masses with vasogenic edema and hemorrhage was a sign of malignancy, which was confirmed with the pathological diagnosis. Diffuse spinal involvement is unusual in this diagnosis, though Rhodes et al. [23] described this finding in one patient.

Treatment options for symptomatic NCM are unsatisfactory. There is little experience in chemo- and radiotherapy, with relatively little effects on the symptoms, and no effects on survival by that care in our patient it were palliative.

Neurocutaneous melanosis is a rare syndrome. The recent data suggest that alteration in hepatocyte growth factor (HGF)/scatter factor (SF) signaling through the* MET* receptor during embryogenesis might play the most important role in the development of NCM. HGF/SF signaling through the* MET* receptor in melanocytes derived from the neural crest promotes the proliferation, motility, and melanin synthesis in vivo [20]. The genetic background of this disorder is not known; therefore genetic counseling is not applicable.

Imaging neither predicts which patients will become symptomatic nor identifies those who might benefit from a proven therapy. Some authors recommend the MRI in the first 4 months of life, as, due to the poor myelinization of CNS, the signal of melanin is easier to detect. Close follow-up with a pediatrician for neurodevelopmental assessment and a dermatologist for skin examination is the best way to address high-risk patients.

## 4. Conclusions

CMN are a diverse group of lesions with significance to life threatening when associated with MM or NCM. Neurological symptoms in patients with NMC may be also related to the development of leptomeningeal melanoma with extremely poor prognosis. A multidisciplinary approach to these patients is imperative and should consider routine neurodevelopmental assessments along with skin examinations for these high-risk patients.
